# Quantitative Proteomic Analysis Provides Novel Insights into Cold Stress Responses in Petunia Seedlings

**DOI:** 10.3389/fpls.2016.00136

**Published:** 2016-02-25

**Authors:** Wei Zhang, Huilin Zhang, Luyun Ning, Bei Li, Manzhu Bao

**Affiliations:** ^1^College of Life Science and Technology, Huazhong Agricultural UniversityWuhan, China; ^2^Key Laboratory of Horticultural Plant Biology, Ministry of Education, College of Horticulture and Forestry Sciences, Huazhong Agricultural UniversityWuhan, China

**Keywords:** cold stress, proteomics, iTRAQ technology, antioxidation mechanism, epigenetic factor, cold-responsive protein, *Petunia hybrida*

## Abstract

Low temperature is a major adverse environmental factor that impairs petunia growth and development. To better understand the molecular mechanisms of cold stress adaptation of petunia plants, a quantitative proteomic analysis using iTRAQ technology was performed to detect the effects of cold stress on protein expression profiles in petunia seedlings which had been subjected to 2°C for 5 days. Of the 2430 proteins whose levels were quantitated, a total of 117 proteins were discovered to be differentially expressed under low temperature stress in comparison to unstressed controls. As an initial study, 44 proteins including well known and novel cold-responsive proteins were successfully annotated. By integrating the results of two independent Gene Ontology (GO) enrichment analyses, seven common GO terms were found of which “oxidation-reduction process” was the most notable for the cold-responsive proteins. By using the subcellular localization tool Plant-mPLoc predictor, as much as 40.2% of the cold-responsive protein group was found to be located within chloroplasts, suggesting that the chloroplast proteome is particularly affected by cold stress. Gene expression analyses of 11 cold-responsive proteins by real time PCR demonstrated that the mRNA levels were not strongly correlated with the respective protein levels. Further activity assay of anti-oxidative enzymes showed different alterations in cold treated petunia seedlings. Our investigation has highlighted the role of antioxidation mechanisms and also epigenetic factors in the regulation of cold stress responses. Our work has provided novel insights into the plant response to cold stress and should facilitate further studies regarding the molecular mechanisms which determine how plant cells cope with environmental perturbation. The data have been deposited to the ProteomeXchange with identifier PXD002189.

## Introduction

Garden petunias (*Petunia hybrida*) are very popular bedding plants around the world. This popularity is due, at least in part, to recent breeding achievements which have combined novel characteristics such as prostrate growth habits with increased robustness in the face of environmental stresses (Griesbach, 2006). However, *P. hybrida* is native to warm habitats, originating from South America. Low temperatures are a crucial limiting factor for the horticultural success of petunia varieties, impacting on their geographical distribution and the length of their display period. Consequently, in northern climates including those of the United States of America, Europe and China, petunia growth is necessarily restricted to environmentally-controlled greenhouses during the late winter and early spring months (Warner and Walworth, 2010), and this inevitably results in considerable expenses for labor and heating. Therefore, a prime target for breeding efforts is the increased cold tolerance of petunia plants. In order to develop sustainable petunia plants cultivated under low temperature conditions, the molecular response of petunia to cold stress needs to be fully understood. This knowledge should identify candidate genes for direct gene manipulation or conventional breeding strategies that will enhance cold hardiness. Groups of differentially expressed regulators of the petunia response at the transcriptional level have previously been described in the context of cold-stress responses, indicating the validity of the transcriptome approach in obtaining meaningful biological information (Li et al., 2015). Nevertheless, a range of studies have demonstrated that transcript levels do not invariably correlate well with the levels of the corresponding proteins (Chen et al., 2002; Tian et al., 2004). This poor correlation is primarily due to the effects of post-translational modifications including ubiquitinylation, phosphorylation, glucosylation and sumoylation (Mann and Jensen, 2003), many of which are pivotal for the regulation of protein function. Therefore, it is necessary to study at the protein level the cellular changes in petunia plants under low temperature stress and, thus, complement the transcriptomic studies in order to further reveal the molecular mechanisms underlying the cellular response to adverse environmental perturbations.

After decades of relatively slow progress, partially because of the greater difficulties encountered in sample preparation of plant tissues, the pace of research into the analysis of protein abundance in plants is beginning to quicken, and this can be attributed to various advancements in proteomic technologies (Thelen and Peck, 2007; Jorrín-Novo et al., 2009). In particular, the translational profiling of diverse plant species under cold stress has attracted much research interest, leading to the identification of differentially expressed proteins (DEPs) which have significantly improved our understanding of the cold response. For example, a proteome study was performed to analyze the cold-stress response of *Arabidopsis* plants by the application of the two-dimensional electrophoresis (2-DE) DIGE technique. The results revealed that, with the approach of proteome, a comprehensive set of proteins related with cellular responses to cold stress could be detected (Amme et al., 2006). Temporal changes in the profile of total proteins in rice leaves after a chilling treatment, and their subsequent recovery, were analyzed based on a 2-D gel electrophoresis technique. From this, 85 DEPs, including many novel cold-responsive proteins were identified; further classification demonstrated that the largest functional category was proteins involved in photosynthesis (Yan et al., 2006). Thus, the study of the influence of cold stress on the proteomes of model plants, cereal crops, woody plants, and other important crop plants, is an actively emerging research area, with publications available for the proteome of *Arabidopsis* (Bae et al., 2003; Amme et al., 2006), rice (Yan et al., 2006; Hashimoto and Komatsu, 2007), spring wheat (Rinalducci et al., 2011), poplar (Renaut et al., 2004), peach (Renaut et al., 2008), pea (Dumont et al., 2011), soybean (Cheng et al., 2010), and chicory (Degand et al., 2009). By contrast, investigations into petunia responses to cold at the protein level are still lacking, thereby restricting our capacity to fully dissect the molecular mechanisms associated with this species' cold stress response.

Traditional 2-DE techniques have been used as the core method in studies to detect the protein expression patterns in diverse plant species. However, in view of certain limitations of these techniques, a number of higher throughput alternatives with improved sensitivity, linearity and reproducibility have been developed and have been applied in plant research. Isobaric tags for relative and absolute quantitation (iTRAQ) coupled to liquid chromatography-quadrupole mass spectrometry (LC-MS/MS) describes a recently developed technique which provides a fast proteomic analytical method for the identification and quantification of expressed proteins with a high degree of efficiency and accuracy (Evans et al., 2012), and is currently being widely used for the quantitative comparative analysis of plant proteomes (Owiti et al., 2011; Zheng et al., 2014). In this study, in order to identify the candidate proteins that are intimately associated with the cold response of petunia, we applied a quantitative proteomic approach combining iTRAQ with LC-MS/MS to detect the DEPs between cold-stressed petunia seedlings and the unstressed controls. Probable biological functions and potential effects of these proteins on cold tolerance are discussed with the aim of determining their roles in cold resistance in petunia. This analysis developed a comprehensive inventory of petunia cold-responsive proteins and highlighted the antioxidation mechanism as well as epigenetic factors in the regulation of the cold stress response.

## Materials and methods

### Plant material, growth conditions, and cold treatment

*Petunia hybrida* inbred line H has been previously described (Li et al., 2015). *In vitro* seedlings of line H were grown in plastic pots at 25°C under long-day conditions (14/10 h light/dark cycle, 2000–2500 lux light intensity) in the laboratory's tissue culture room for 1 month. Plants of a uniform growth status at the developmental stage 4–5 pairs of true leaves were subsequently transferred to the cold-stress conditions (2°C, 500–1000 lux light intensity). After 5 days of treatment, the stressed plants were harvested and immediately frozen in liquid nitrogen, and then held at −80°C until required for further processing. Untreated plants (0 h cold stress) were used as controls. Four individual plants were harvested and pooled for each sample, and this collection was repeated four times to provide biological replicates.

### Protein extraction and digestion

Seedlings from cold-treated plants and control plants were ground into powder with liquid nitrogen and suspended in a 10 × volume of pre-cooled acetone (−20°C) containing 10% (v/v) TCA. After thorough mixing, proteins were precipitated at −20°C overnight. Proteins were then collected by centrifuging at 10,000 rpm (Eppendorf5430R; Eppendorf Ltd., Hamburg, Germany) at 4°C for 45 min. The supernatant was carefully removed, and the protein pellets were washed twice with cold acetone. Protein pellets were dried by lyophilization and then extracted using a 10 × volume of SDT buffer composed of 4% (v/v) sodium dodecyl sulfate (SDS) (Bio-Rad, Hercules, CA, USA), 1 mM dithiothreitol (DTT) (Bio-Rad, Hercules, CA, USA) and 150 mM TrisHCl (pH 8.0), with incubation in a boiling water bath for 5 min. Protein extracts were subsequently dispersed by ultrasonication (80 w: 10 times for 10 s each, with 15 s intervals in between). After heating in a boiling water bath for 5 min, the final protein pellets were obtained by passing the extract through a filter tube (0.22 μm diameter). The resulting protein concentration was determined using BCA Protein Assay Kit (Pierce, Thermo Scientific, Rockford, IL, USA).

For each sample, 300 μg of proteins were incorporated into 30 μL of STD buffer, composed of 4% (v/v) SDS, 100 mM DTT and 150 mM Tris-HCl (pH 8.0). Removal of DTT and other low-molecular-weight components was achieved by repetitive ultrafiltration using UA buffer composed of 8 M Urea (Bio-Rad, Hercules, CA, USA) and 150 mM TrisHCl (pH8.0). Subsequently, by adding 100 μL of 0.05 M iodoacetamide (IAA) (Bio-Rad, Hercules, CA, USA) in UA buffer, the samples were incubated in darkness for 20 min in order to block the reduced cysteine residues. To wash the filters we used 100 μL of UA buffer (three times), followed by 100 μL of DS buffer (50 mM triethylammonium bicarbonate pH 8.5) (two times). Finally, 2 μg trypsin (Promega, Madison, WI, USA) in 40 μL of DS buffer was used to digest the protein suspensions by incubation at 37 °C overnight, and the digested peptides were collected as a filtrate. An extinction coefficient of 1.1 (0.1% g/L solution, calculation based on the frequency of tryptophan and tyrosine in vertebrate proteins) was used to evaluate the peptide content by UV light spectral density at 280 nm.

### iTRAQ labeling and peptide fractionation

Peptide samples were labeled with 8-plex iTRAQ reagents (Applied Biosysterms) according to the manufacturer's protocol. Four samples from cold-treated seedlings were labeled with reagent 113, 114, 115, and 116, respectively. Four control samples from untreated seedlings were labeled with reagent 117, 118, 119, and 121, respectively.

iTRAQ labeled peptides were combined and further fractionated with the AKTA Purifier system (GE Healthcare) by strong cation exchange (SCX) chromatography. In brief, the dried peptide mixtures were reconstituted and acidified with buffer A (10 mM KH_2_PO_4_ in 25% of ACN pH 3.0), then, loaded onto a polysulfethyl 4.6 × 100 mm column (5 μm, 200 Å) (PolyLCInc, Maryland, U.S.A.). The peptides were eluted with a gradient buffer B (10 mM KH_2_PO_4_, 500 mM KCl in 25% of ACN pH 3.0) (0-10% for 7 min, 10-20% for 10 min, 20-45% for 5 min, and 45-100% for 5 min) at a flow rate of 1 mL/min. The absorbance at 214 nm was monitored and a total of 10 final fractions were collected. Each final fraction was desalted on C18 cartridges (Sigma, Gillingham, UK) and concentrated by vacuum centrifugation. All samples were stored at −80°C.

### LC-MS/MS measurement

The peptide mixtures were loaded onto a packed capillary tip (C18-reversed phase column with 15 cm long, 75 μm inner diameter) with RP-C18 5 μm resin, washed in buffer A (0.1% formic acid), and subsequently separated with a linear gradient of buffer B (0.1% formic acid and 84% acetonitrile) at a flow rate of 250 nL/min over 120 min: 0–100 min with 0–45% buffer B; 100−108 min with 45–100% buffer B; 108–120 min with 100% buffer B. The Q-Exactive (Thermo Finnigan, San Jose, CA, USA) mass spectrometer was used to acquire data in the positive ion mode, with a selected mass range of 300–1800 mass/charge (m/z). Survey scans were acquired at a resolution of 70,000 at m/z 200, and the resolution for HCD spectra was set as 17,500 at m/z 200; MS/MS data were acquired using a data-dependent “top10” method to capture the most abundant precursor ions. The normalized collision energy was 30 eV; the underfill ratio was defined as 0.1% on the Q-Exactive; and the dynamic exclusion duration was 40 s.

### Protein identification and quantification

Protein identification and quantification were simultaneously performed with MASCOT 2.2 (Matrix Science, London, U.K.) embedded into Proteome Discoverer 1.4 (Thermo Electron, San Jose, CA, USA), searching against the Uniport database of combined protein sequences of solanaceae, *Solanum lycopersicum* and *Solanum tuberosum* (uniprot_solanaceae_108653_20130709.fasta, uniprot_Solanum lycopersicum_36345_20130710.fasta, uniprot_Solanum tuberosum_55352_20130710.fasta, downloaded from: http://www.uniprot.org/) and the decoy database. Search parameters were set as follows: trypsin as the enzyme; monoisotopic mass; a permitted maximum of two missed cleavages; peptide mass tolerance at ±20 ppm and fragment mass tolerance at 0.1 Da. Lysine and N-term of peptides labeled by iTRAQ 8-plex and carbamidomethylation on cysteine were specified as fixed modifications, while variable modifications were defined as oxidation of methionine and iTRAQ 8-plex labeled tyrosine. False discovery rate (FDR) of both proteins and peptides identification was set as: FDR ≤ 1%. Protein identifications were supported by a minimum of one unique peptide identification.

### DEPs identification, annotation and subcellular localization prediction

The normalization of the ratios for the iTRAQ labels was performed according to the user's guide of the Proteome Discoverer (Version 1.3). The final ratios of proteins were normalized by the median average protein ratio of the equal mix of different labeled samples. iTRAQ ratios were log-transformed before being analyzed mathematically. Only proteins detected in all runs (every biological replicate) were included in the data set. To identify the DEPs, the “t.test” function in R program (http://www.r-project.org/) with default settings (alternative = “two.sided,” var.equal = FALSE) were used to calculate the *P*-values of the students' *t*-Test, and the *P* < 0.05 was applied. The higher average in cold stressed plant than control was labeled as up-regulated proteins, and the lower in treatment group was regarded as down-regulated. Differentially abundant proteins were further functionally annotated using Blast2Go. GO enrichment analysis was performed using the singular enrichment analysis (SEA) under agriGO toolkit (Du et al., 2010), and the *Arabidopsis thaliana* (TAIR9) as well as *Solanum lycopersicum* (Tomato Affymetrix array) were used as backgrounds in combination with Fisher's test and Yekutieli multiple-test with a threshold of FDR = 0.05.

Using the tool of Plant-mPLoc predictor, prediction of subcellular localizations of DEPs was performed. Plant-mPLoc is becoming widely used for the prediction of plant protein subcellular localization as it has the capacity to deal with multiple-location proteins, which is beyond the capability of other existing predictors specialized for identifying plant protein subcellular localization (Chou and Shen, 2010).

### Transcriptional validation by real-time PCR analysis

Total RNA was extracted from whole plantlets (taken from the same treatment samples as used for protein extraction) by using the EASYspin Plant RNA Mini kit according to the manufacturer's protocol (Aidlab, Beijing, China). RNA concentration and integrity estimation, reverse transcription, and real-time PCR were performed according to previous descriptions (Li et al., 2015). The primers were designed according to the corresponding nucleotide sequences of *Petunia hybrida* in GenBank. Gene-specific primers for real-time PCR analysis are presented in Table S1 in the Supplementary Material.

### Activity assay of anti-oxidative enzymes

Three hundred milligram fresh leaves were frozen in liquid nitrogen and then ground in 3 ml solution containing 100 mM phosphate buffer (pH 7.8) and 1% (w/v) polyvinylpolypyrrolidone. The homogenate was centrifuged at 3500 rpm for 15 min, and the supernatant was collected for enzyme assays. All operations above (until analysis) were carried out at 4°C. The enzyme activities of catalase (CAT), superoxide dismutase (SOD), and glutathione peroxidase (GPX) were determined by using the kits according to the manufacturer's protocol (Nanjing Jiancheng Institute of Biotechnology, China).

## Results

### Genome-wide proteomics identification and evaluation

In order to investigate the proteomic changes associated with petunias exposed to a low temperature treatment (2°C), iTRAQ analysis was conducted to compare the DEPs between the control and cold-treated plants. In this study, we used high accuracy LC–MS/MS to quantitatively detect and map proteins in the petunia seedlings. The protein concentration of samples was determined by BCA (Table S2). For the purposes of quality control, 20 μg protein aliquots from each sample were evaluated by SDS-PAGE analysis (Figure S1). The abundance of digested peptides was quantified based on UV-absorption at 280 nm (Table S3). The combined iTRAQ labeled peptides were fractionated by strong cation exchange (SCX) chromatography (Figure S2). The mass spectrometry proteomic data of the present study have been deposited to the proteomics data repository - PRIDE Archive (http://www.ebi.ac.uk/pride). Project Accession: PXD002189; http://www.ebi.ac.uk/pride/archive/projects/PXD002189.

**Figure 1:**
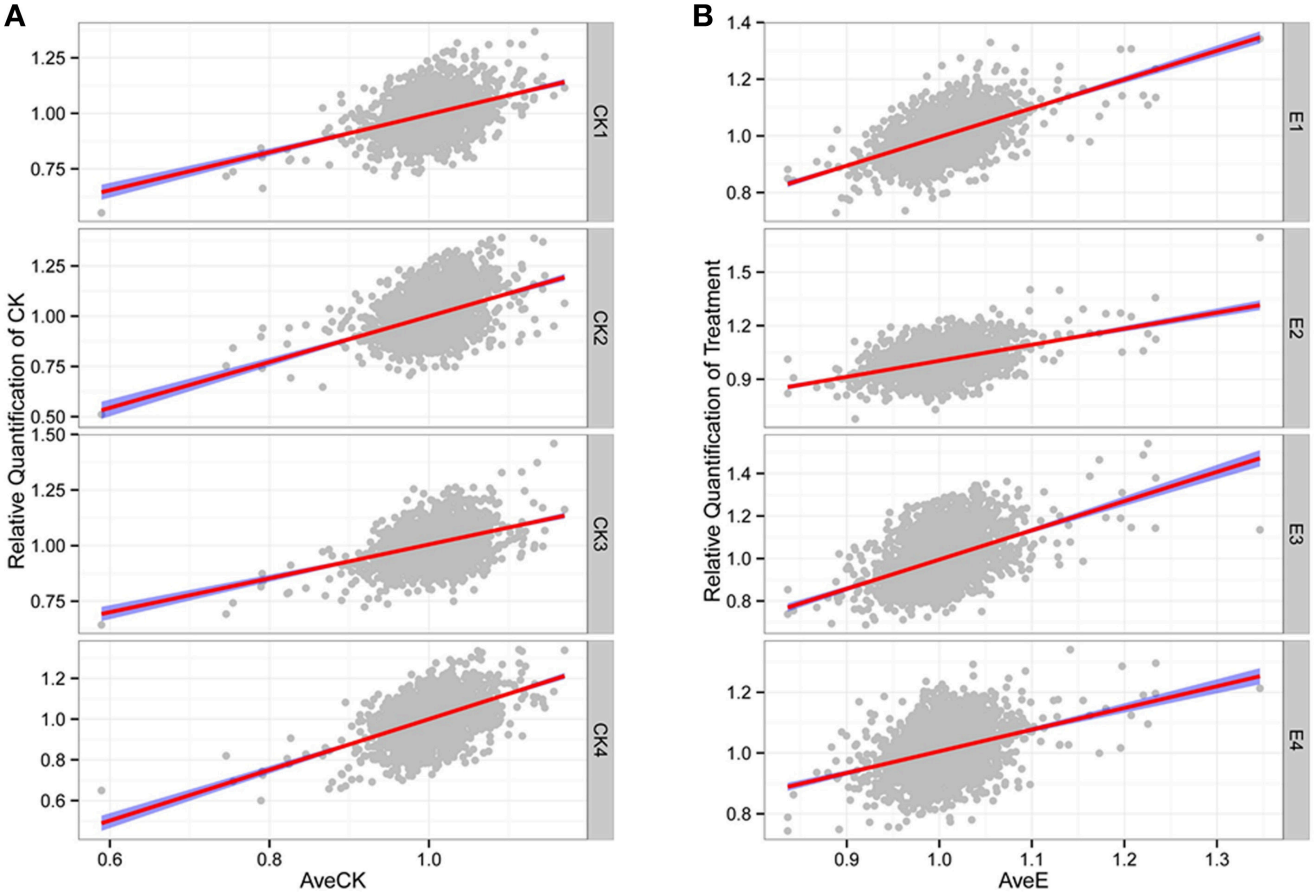
**High correlation among different replicates. (A)** Relative quantification of CK. **(B)** Relative quantification of treatment. The “AveCK” represents the average quantification among control samples, and the “AveE” means the average quantification among experimental samples, and the “CK1~4” and “E1~4” are the corresponding different individuals.

A total of 8066 unique peptides (FDR < = 0.01) were obtained (Table S7) and 2862 proteins were ultimately identified (Table S8). Amongst all of the detected proteins, 2430 common proteins were detected in each replicate of all samples, and their relative quantifications (Table S6) were used for further analyses. The relative abundance levels within each group showed high degrees of positive correlation (*P* < 2.2E-16; Figure 1), thereby indicating that the overall experimental process and the quantification methods were reliable. By contrast, the genome-wide protein abundance in each sample was highly variable (Figure S3; Table S6), so illustrating the complexity of the regulatory picture in active cells. Thus, as expected, the predicted molecular weights and pIs of the various identified proteins also showed high degrees of variation (Table S6), with molecular weights ranging from 1.19 to 1445.78 kDa with a median of 37.72 kDa, and pIs ranging from 3.92 to 12.38 with a median of 6.8. Together, these results revealed an abundance of diverse proteins in the sampled petunia seedlings and confirmed the effectiveness of the high-throughput methodology used in this study.

### Identification, functional annotation and subcellular localization of DEPs

Since the draft genome sequence of petunia was not publicly available at the time of this study, we used other closely related species that had already been sequenced, such as *Solanum lycopersicum*, in order to annotate all of the identified proteins. Statistical *t*-test analysis was used to identify the possible candidate proteins that are involved in the petunia cold stress response. Differential expression data are summarized in Table S6. Of the 2430 proteins that were quantitated, a total of 117 unique proteins showed differential expression whereas, 2313 proteins were unchanged by cold stress or did not meet the criteria for statistical significance. Of these 117 DEPs, 67 were found to be up-regulated, with the other 50 DEPs down-regulated in treatment lines when compared with the controls. Although a large portion of DEPs were found to either share homology with putative proteins of unknown function or shared no significant homology with any of the database accessions, the remaining 44 identified proteins were successfully annotated and are listed in Table 1. GO enrichment analyses of the cold-responsive DEPs against the genome-wide databases of tomato and *Arabidopsis* (Table S7) showed enrichment for 20 and 18 GO terms, respectively. These enriched groups included various biological processes and molecular functions, including cellular biosynthetic process, various binding activities and catalytic activities, and major cellular components integral to membranes. Notably, seven GO terms were found to be common to the results of these two independent GO enrichment analyses (Table 2), including the terms: oxidation reduction, cation binding, ion binding and intrinsic to membrane etc. These conserved enriched groups offer insights into the biological pathways important to the petunia response to cold stress.

**Table 1:** **Information of DEPs**.

**Accession**	**Description**	**Variation in protein relative abundance**	***P*-value**
B5LAT1	Putative ketol-acid reductoisomerase	Down	0.0120
B7ST71	Glutathione-dependent formaldehyde dehydrogenase	Down	0.0142
E2FAU0	COSII_At5g14320	Down	0.0490
K4ASP6	Methionine aminopeptidase	Down	0.0491
K4CUX6	Elongation factor Tu	Down	0.0132
M1B641	Elongation factor Tu	Down	0.0120
M1BC44	Beta-hexosaminidase	Down	0.0011
M1BEC3	Histone H3	Down	0.0206
M1D6S9	Ribosomal protein L15	Down	0.0323
P21342	Pyrophosphate–fructose 6-phosphate 1-phosphotransferase subunit alpha	Down	0.0466
Q2LFC1	AGO4-2	Down	0.0471
Q2XPW5	40S ribosomal protein S3a	Down	0.0311
Q32ZI8	PEN2-like protein	Down	0.0450
Q3HRW8	60S ribosomal protein L18a	Down	0.0371
Q40878	P17	Down	0.0006
Q43779	Superoxide dismutase [Cu-Zn] 2	Down	0.0170
Q5BN14	Pyruvate decarboxylase 1	Down	0.0159
Q5EEY5	SGT1	Down	0.0448
Q84UY3	Alcohol dehydrogenase 2	Down	0.0446
Q94I89	Putative NtPRp27-like protein	Down	0.0223
B6EWX5	UDP-glucose:glucosyltransferase	Up	0.0303
E2FAX1	COSII_At5g14320	Up	0.0005
E5F371	Dehydrin	Up	0.0064
I7J2T7	Ran GTPase activating protein 2	Up	0.0427
K4CW69	Cyanate hydratase	Up	0.0173
M1APP3	ATP-dependent Clp protease proteolytic subunit	Up	0.0288
M1AWZ7	Glutathione peroxidase	Up	0.0075
M1C0V6	Fructose-bisphosphate aldolase	Up	0.0097
M1CXI6	Aspartate aminotransferase	Up	0.0108
M1D227	Carbonic anhydrase	Up	0.0352
P30360	Probable cinnamyl alcohol dehydrogenase 2	Up	0.0165
P32111	Probable glutathione S-transferase	Up	0.0458
P48498	S-adenosylmethionine synthase 1	Up	0.0241
P61046	Cytochrome b6-f complex subunit 8	Up	0.0316
Q07346	Glutamate decarboxylase	Up	0.0145
Q1A531	Phospholipase C	Up	0.0476
Q3LHL1	Short chain dehydrogenase	Up	0.0125
Q6T2D5	Catalase	Up	0.0181
Q8S346	Putative expansin	Up	0.0183
Q94IK0	Ferredoxin-thioredoxin-reductase catalytic subunit B	Up	0.0308
Q9AWA9	Non-symbiotic hemoglobin class 1	Up	0.0124
Q9FEB8	Acetolactate synthase	Up	0.0301
Q9FXS3	Probable phospholipid hydroperoxide glutathione peroxidase	Up	0.0118
Q9ZWH9	Elongation factor 1-alpha	Up	0.0375

**Table 2:** **Common GO terms by integration of the results of two independent GO enrichment analyses**.

**GO term**	**Ontology**	**Description**	**p(*Arabidopsis*)**	**FDR(*Arabidopsis*)**	**p(tomato)**	**FDR(tomato)**
GO:0055114	BP	Oxidation reduction process	2.30E-07	9.60E-05	7.00E-09	6.50E-08
GO:0043169	MF	Cation binding	0.00032	0.02	0.00024	0.00088
GO:0043167	MF	Ion binding	0.00032	0.02	0.00024	0.00088
GO:0016021	CC	Integral component to membrane	2.90E-12	2.90E-10	1.70E-09	3.10E-08
GO:0031224	CC	Intrinsic component to membrane	1.40E-09	7.30E-08	4.20E-09	3.80E-08
GO:0044425	CC	Membrane part	1.30E-07	4.20E-06	5.60E-08	3.40E-07
GO:0016020	CC	Membrane	0.00015	0.0037	8.70E-06	4.00E-05

The subcellular localizations of the identified cold-responsive proteins were determined using the Plant-mPLoc predictor (Chou and Shen, 2010). The results of these predictions showed that DEPs were typically located in various organelles such as chloroplasts, the nucleus, peroxisomes, Golgi apparatus and also in the cytoplasm. Notably, as many as 47/117 (i.e., 40.2%) of the cold-responsive proteins were predicted to be targeted to chloroplasts (Table S8), and these 47 proteins were comprised of 19 down-regulated proteins (i.e., 40.4%) and 28 up-regulated proteins (i.e., 59.6%). This finding suggests that chloroplasts are significant cellular organelles with regard to the cold stress response in petunia.

### Comparison between mRNA and protein levels of selected proteins

**Figure 2:**
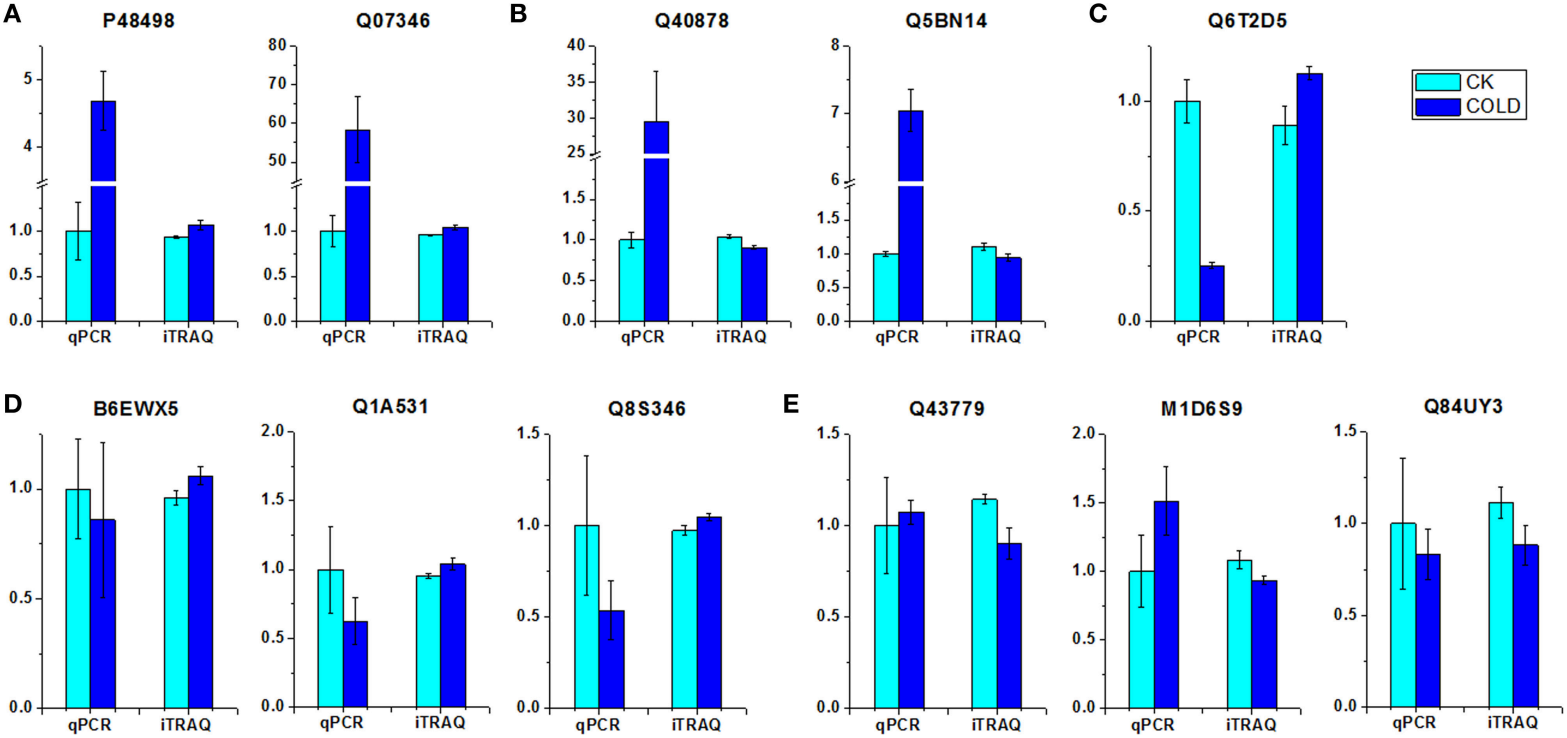
**Comparison of expression patterns at the mRNA and protein level of DEPs. (A)** Up-regulated at both transcript and protein level. **(B)** Up-regulated at transcript level while down-regulated at protein level. **(C)** Down-regulated at transcript level while up-regulated at protein level. **(D)** No change at transcript level while up-regulated at protein level. **(E)** No change at transcript level while down-regulated at protein level. Relative transcript levels were calculated by real-time PCR with *GAPDH* as a standard.

To investigate the transcript levels of DEPs, real-time PCR analysis was performed using the same plant materials as employed for iTRAQ. Eleven proteins, either up- or down-regulated under cold stress, were selected for the design of gene-specific primers (Table S1) for use in real-time PCR. Of the selected proteins, nine different enzymes such as superoxide dismutase (Q43779), alcohol dehydrogenase 2 (Q84UY3) and pyruvate decarboxylase 1 (Q5BN14), and also one ribosomal protein (M1D6S9) and one putative expansin (Q8S346), were included. The results of real-time PCR demonstrated that only two genes (corresponding to P48498 and Q07346) displayed accordant change tendency as the results of iTRAQ. By contrast, six genes (corresponding to B6EWX5, Q1A531, Q43779, M1D6S9, Q84UY3, and Q8S346) showed no significant changes at the transcript level, despite the detection of differential expression patterns at the protein level, as indicated by iTRAQ data. Interestingly, the remaining three genes (corresponding to Q40878, Q5BN14, and Q6T2D5) showed completely contrary trends between transcriptome and proteome levels. On the basis of these patterns of association between the mRNA and protein levels, the eleven selected DEPs could be clustered into five groups (Figure 2), i.e., group I, up-regulated at both transcript and protein levels (Figure 2A); group II, up-regulated at transcript level while down-regulated at protein level (Figure 2B); group III, down-regulated at transcript level while up-regulated at protein level (Figure 2C); group IV, no change at transcript level while up-regulated at protein level (Figure 2D); group V, no change at transcript level while down-regulated at protein level (Figure 2E). These analyses showed that both parallel and independent correlations existed between the mRNA and protein expression profiles among cold-responsive proteins, which indicates the existence of a highly complex regulatory network in petunia seedlings exposed to cold.

### Effect of cold stress on anti-oxidative enzymes

**Figure 3:**
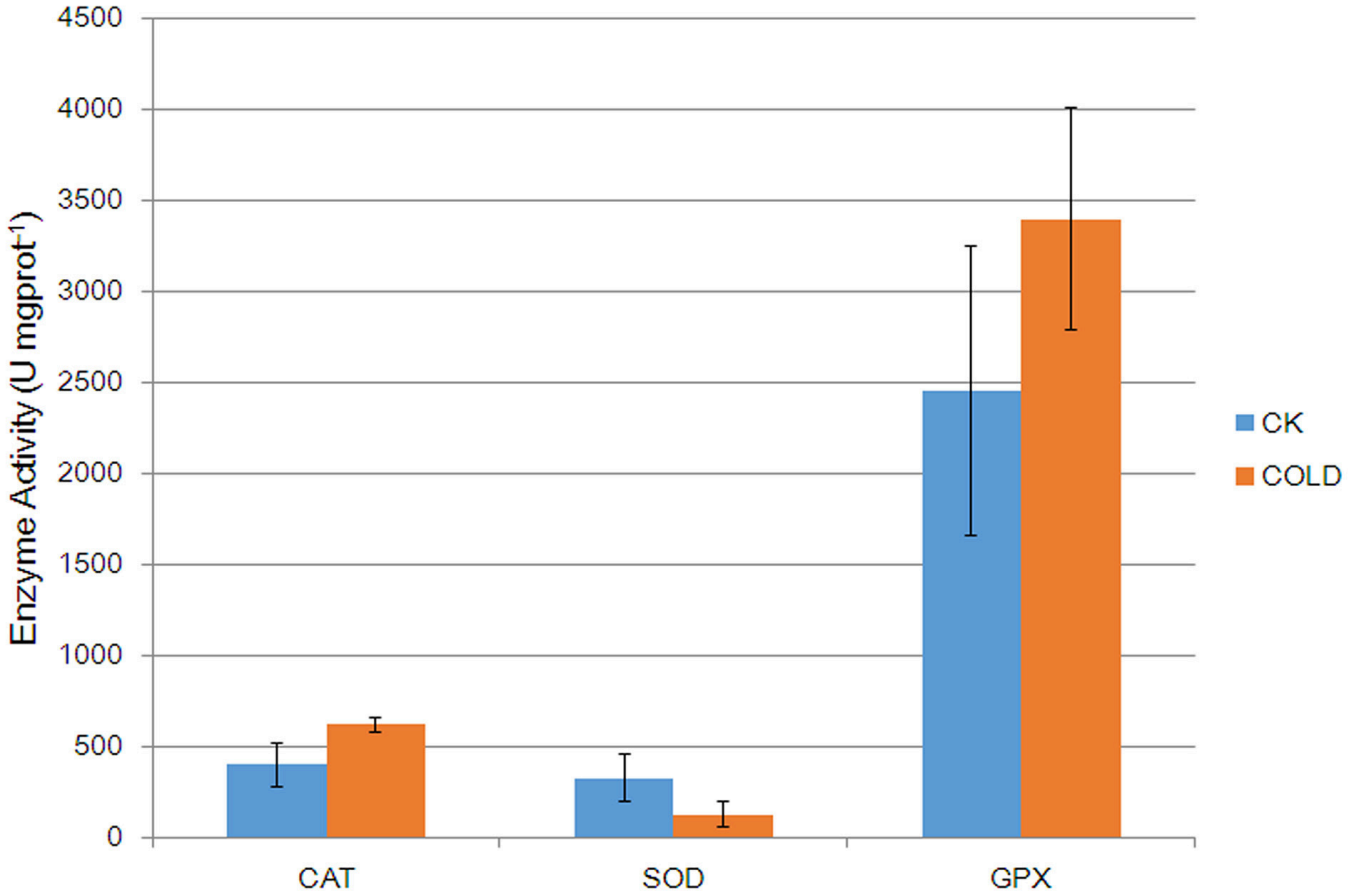
**Catalytic activities of anti-oxidative enzymes in control plants and cold-treated plants**. Columns and bars represent the means and SE (*n* = 3), respectively.

Five anti-oxidative enzymes were affected by cold stress (Table 1). In order to gain more in-depth insights into the change of anti-oxidative enzymes under cold, activities of three anti-oxidative enzymes (CAT, SOD, and GPX) were investigated (Figure 3). Results showed that the activities of CAT and GPX were increased, while SOD activity was decreased in cold treated petunia seedlings, further suggesting association between antioxidation mechanisms and cold stress response in petunia.

## Discussion

Our transcriptome analyses have identified several candidate genes which may be involved in the cold stress response in petunia plants (Li et al., 2015). This initial proteomic analysis of petunia seedlings identified several cold-responsive proteins and revealed a complex cellular network affected by the cold stress treatment. Perhaps unsurprisingly, these candidate proteins included some with previously well recognized roles as general stress-inducible proteins, such as catalase and dehydrin. In addition, some of the petunia cold-responsive proteins identified here have been previously verified in other plants using the proteomic approach to cold stress; these proteins include carbonic anhydrase (Gao et al., 2009), beta-hexosaminidase (Yang et al., 2012), and fructose-bisphosphate aldolase (Yang et al., 2012), etc. Furthermore, some novel proteins were also identified in the petunia cold response. These results have demonstrated that, by applying the iTRAQ technology, a comprehensive set of proteins correlated with cellular responses to cold stress in petunia can be detected. These findings support the reliability and robustness of the iTRAQ approach for determining differentially regulated protein responses. The possible biological significance of key DEPs in cold stress adaptation and their associated metabolic pathways are discussed below.

### Association between antioxidation mechanisms and cold stress response in petunia

By integrating the results of two independent GO functional enrichment analyses, the petunia cold-responsive proteins were found to be enriched for seven common GO terms (Table 2). Of these common terms, “oxidation-reduction process” was the most notable, from which we tentatively suggest that antioxidation mechanisms may contribute to the adaptive response to low temperatures in petunia plants. These findings are consistent with the current understanding that a single environmental stress may simultaneously trigger multiple stress responses at an intracellular level. Cold stress, along with other abiotic stress types, is known to induce the production of reactive oxygen species (ROS). These can perturb cellular redox homeostasis and result in oxidative damage to membrane lipids, proteins and nucleic acids, ultimately leading to stress injuries in plants. To counterbalance this ROS accumulation, plants subjected to cold stress conditions can induce and activate scavenging systems, and trigger the expression of proteins able to protect cell machinery.

For example, detoxifying enzymes such as CATs and SODs are induced by cold stress in *Arabidopsis* and rice (Goulas et al., 2006; Guo et al., 2006). Likewise, we observed higher levels of one petunia CAT (Q6T2D5) which was predicted to be located in the peroxisome (Table S8). In contrast, one molecular form of SOD, i.e., Cu/Zn-SOD (Q43779), was down-regulated by cold stress. Because the altered patterns of CAT and Cu/Zn-SOD at the mRNA and protein levels were not consistent under cold stress (Figure 2), it is suggested that these two enzymes are possibly regulated by post-transcriptional mechanisms. SODs catalyze the dismutation of superoxides into O_2_ and H_2_O_2_ (Apel and Hirt, 2004). Although SODs are recognized as general stress-inducible proteins, the effect of cold on the expression of Cu/Zn-SOD in this current study was actually not a surprise. It was reported that Cu/Zn-SOD was down-regulated in rice leaf sheaths exposed to 5°C (Hashimoto and Komatsu, 2007). Similarly, a decrease in levels of Cu/Zn-SOD was found in strawberry plants after a cold treatment (Koehler et al., 2012). The accord of these published results together with our own findings suggests specificity of certain Cu/Zn-SODs in the plant response to cold stress. CAT, which is mainly localized within peroxisomes, catalyzes the decomposition of H_2_O_2_ to oxygen and water via the CAT pathway. Our results are consistent with previous studies and further implicate CAT in the response to low temperature, thereby prompting us to speculate that ROS scavenging through the effects of the CAT pathway may contribute to the adaptation of petunia plants coping with adverse ambient conditions. More recently, in addition to those well characterized antioxidant enzymes such as CATs and SODs, the role of glutathione S-transferase (GST) and GPX during various stress conditions in plants has been reported by an increasing number of publications. For instance, overexpression of a cDNA encoding an enzyme with both GST and GPX activity has been reported to enhance the growth of transgenic tobacco seedlings under cold and salt stresses (Roxas et al., 1997). GSTs catalyze the conjugation of glutathione (GSH) to a wide variety of hydrophobic and electrophilic compounds to form non-toxic, or at least less toxic, peptide derivatives (Marrs, 1996; Frova, 2003). In addition, diverse isoforms of GSTs isolated from different plant species also showed significant GPX activity toward lipid hydroperoxides, catalyzing their reduction to the less toxic alcohols (Bartling et al., 1993; Cummins et al., 1999). GPXs are ubiquitously occurring enzymes in plant cells which use GSH to reduce H_2_O_2_ and organic and lipid hydroperoxides (Milla et al., 2003; Navrot et al., 2006). Therefore, it was not surprising that both GST (P32111) and GPX (M1AWZ7) were up-regulated in petunia seedlings exposed to cold. We assume that GST and GPX activities are involved in the alterations of GSH and ascorbate metabolism that lead to reduced oxidative damage and enhanced tolerance to stresses, alongside the scavenging of peroxides (Roxas et al., 2000). Moreover, phospholipid hydroperoxide glutathione peroxidase (PHGPx, Q9FXS3) was also up-regulated at the protein level. PHGPx is a unique antioxidant enzyme responsible for reducing lipid hydroperoxides directly, which is generally considered the principal enzymatic defense against oxidative biomembrane destruction in animals (Imai and Nakagawa, 2003). In plants, however, the role of PHGPx has so far remained largely unexplored. Investigations of tissue expression and induction expression profiles at the protein level under a wide range of abiotic stresses (Li et al., 2009) have highlighted the likelihood of a specific role for PHGPx in ROS scavenging. Our results suggest that the GSH cycle enzymes might also play a significant role as part of an antioxidant protection system in the petunia response to cold stress. Furthermore, activity assay confirmed that anti-oxidative enzymes were regulated in cold treated petunia seedlings (Figure 3), suggesting that they also suffered from oxidative stress. Taken together, these results linked antioxidation mechanisms with cold stress response.

### Epigenetic factors involved in cold stress response of petunia

In order to adapt to environmental challenges, it is of great importance for sessile plants to dynamically control gene expression patterns. This is particularly vital for stress responses, which are controlled through a myriad of signal transduction pathways. For example, when environmental cues are perceived and transmitted, specific transcription factors in the nucleus are turned on and a cascade of downstream gene expressions is triggered. In recent years, it has become evident that the biogenesis of small RNAs and dynamic changes in chromatin properties also contribute to the regulation of gene expression. Studies have indicated that these epigenetic mechanisms are crucial to appropriate plant reactions to stress (Borsani et al., 2005; Angers et al., 2010; Kumar and Wigge, 2010). In our study, the abundance of two epigenetic factors namely, the histone H3 (M1BEC3) and a member of the Argonaute protein family AGO4-2 (Q2LFC1), were decreased after cold stress. It was previously reported that histone H1 was up-regulated at the transcript level in *Arabidopsis* in response to cold-, salt- and drought-stress (Kreps et al., 2002). Recently, a quantitative proteomic analysis in rice has revealed several cold-responsive histones. Among them, H4, H2B.9, H3.2, and linker histones H1 and H5, were found to be down-regulated (Neilson et al., 2011). Histones are prone to reversible post-translational modifications such as acetylation, methylation, phosphorylation, ubiquitination, and glycosylation, which allow the proteins to respond flexibly to stimuli (Neilson et al., 2011). In rice, submergence of the seedlings under water induced histone H3 acetylation and H3K4 trimethylation in pyruvate decarboxylase 1 (*PDC1*) and alcohol dehydrogenase 1 (*ADH1*) genes. These histone modifications were associated with enhanced expression of *PDC1* and *ADH1* at the transcript level under stress (Tsuji et al., 2006). In fact, histone modification is a critical regulator of gene expression and has been implicated in plant stress responses, including the response to low temperature (Zhu et al., 2008; Kim et al., 2010a). Intriguingly, our proteomic data showed that cold stress also affected the expression levels of PDC1 (Q5BN14) and ADH2 (Q84UY3). It is not clear whether there was any direct relationship between the varied expression pattern of histone H3 and that of PDC1 or ADH in this study; however, a quantitative study of histone post-translational modifications could provide valuable information as to the role in the regulation of cold hardiness. AGO4 is one of the crucial components in the transcriptional gene-silencing pathway correlated with siRNA which directs DNA methylation at specific loci, a phenomenon referred to as RNA-directed DNA methylation (RdDM) (Agorio and Vera, 2007). As a small RNA biogenesis factor that is involved in the biogenesis of heterochromatic siRNAs (hc-siRNAs) and in the pathway of RdDM, AGO4 is essential for antibacterial resistance; in addition, it plays an important role in plant resistance to viruses (Agorio and Vera, 2007; Bhattacharjee et al., 2009). As shown by our iTRAQ data, AGO4-2 expression was altered in response to low temperature, which would offer the possibility that AGO4 is also involved in defensive reactions against abiotic stresses. Since plant epigenetics has recently attracted unprecedented interest, not only as a subject of basic research but also as a potential new source of advantageous characters for plant breeding (Mirouze and Paszkowski, 2011), the identification of these two epigenetic regulators in this work might suggest a new direction for research into the cold tolerance of petunia.

### Other cold responsive proteins in petunia

Several DEPs identified in this study were predicted to be involved in antioxidative/detoxifying reactions and epigenetic regulation, as discussed above, whereas others that formed the focus of this study were associated with several primary and secondary metabolic processes, such as protein synthesis, energy metabolism and phenylpropanoid biosynthesis.

Six DEPs, including three ribosomal proteins (RPs) and three elongation factors, were related to protein synthesis. RPs are essential for protein synthesis and have been revealed to play an important role in metabolism, cell division and growth (Wang et al., 2013). Besides their housekeeping functions, it is interesting to note that there is an increasing awareness of the function of some RPs in other roles. For instance, ribosomal protein S3 (RPS3), a component of the eukaryotic 40S ribosomal subunit, has been proposed to play a central role in regulating numerous aspects of host–pathogen interactions (Gao and Hardwidge, 2011). Although to date there is little evidence for direct links between RPs and cold stress, we shouldn't ignore the decreased levels of three RPs (Q2XPW5, Q3HRW8, and M1D6S9) observed in our experiments. We speculate that RPs participate as regulatory components in the response to stress, although the regulation mechanism remains to be elucidated. Elongation factor Tu (EF-Tu) is an organelle protein playing a central role in the elongation phase of protein synthesis. EF-Tu gene expression has been extensively studied in plants in response to various environmental challenges, especially high temperature stress (Bhadula et al., 2001; Bukovnik et al., 2009). Moreover, there is growing evidence regarding abiotic stress-related EF-Tu expression, which has been acquired using proteomics approaches. For example, proteomic analyses of cold- and heat-stress responses in rice identified plastid EF-Tu as an up-regulated protein (Cui et al., 2005; Lee et al., 2007). On the contrary, in the present work we found two EF-Tu proteins (K4CUX6 and M1B641) that displayed decreased levels in cold-treated petunia seedlings, although a rationale for these findings couldn't be deduced yet. Nevertheless, EF-1α (Q9ZWH9), the cytosolic homolog of EF-Tu in plants which is also pivotal in the regulation of translation under abiotic stresses, was present at a higher level in seedlings at 2°C. The differential regulation of various components of the translation machinery implies that a complicated mechanism governing protein synthesis exists in response to cold stress. In addition to their roles in translation, within bacteria, mammalian cells and in plants such as *Arabidopsis*, EF-Tu and EF-1α seem to display chaperone-like activities in protein folding, in protection against thermal denaturation and in interaction with unfolded proteins (Suzuki et al., 2007; Shin et al., 2009). We tentatively suggest that these factors, which are implicated in cold adaptation, may also have similar functions in petunia.

Among the cold responsive proteins, three proteins were identified to be energy-related enzymes, including cyanate hydratase (K4CW69), carbonic anhydrase (M1D227) and ATP-dependent Clp protease proteolytic subunit (M1APP3). The functions of these enzymes under cold stress conditions are still unknown. Cyanate hydratase, which catalyzes the bicarbonate-dependent breakdown of cyanate to ammonia and bicarbonate in cyanogenic glycosides, was induced by cold stress. Along with earlier results from tomato and grapevine (Parker et al., 2013; Liu et al., 2014), a probable role was suggested for cyanate hydratase in plant responses to both biotic and abiotic stresses. A chloroplast-localized carbonic anhydrase, which facilitates CO_2_ movement across the chloroplast envelope, was found to decrease in abundance in *Thellungiella* rosette leaves after 5 days of cold treatment (Gao et al., 2009). However, this enzyme, which was predicted to be also localized in the chloroplasts (Table S8), showed the opposite expression pattern in our experiments. The different results were likely due to the distinctness of plant materials and differences in experimental conditions. ATP-dependent Clp protease proteolytic subunit was shown to decrease in abundance in cultured rice cells at 44°C (Gammulla et al., 2010). In such a context, our finding that ATP-dependent Clp protease proteolytic subunit increased at 2°C further supported its involvement in the regulation of temperature stress response.

Two DEPs correlated with the plant cell wall (CW) were found to be induced by cold stress. One was a cinnamyl alcohol dehydrogenase (CAD) (P30360) and the other was a putative expansin (Q8S346). The plant CW forms a barrier against pathogen attack and interconnects cells, and thereby plays a variety of distinct, sometimes opposite, roles. It is interesting to note that the CW may play a critical role in plant cold resistance (Tao et al., 1983). Pronounced thickening of CW has been observed in cold-acclimated plants of diverse species, suggesting an increased lignin production during cold acclimation. Lignin is a class of complex organic polymers of phenylpropanoid compounds and is particularly important in the formation of the plant CW. It is believed that CW thickening could provide resistance against cell collapse and, thus, may provide protection against mechanical stresses induced by cold (Wei et al., 2006). CAD is a major rate-limiting enzyme that catalyzes the final step of the lignin biosynthesis, the conversion of cinnamyl aldehydes to alcohols, with NADPH acting as the cofactor (Sattler et al., 2010). While CAD is multifunctional, what interests us is the relationship between the CAD gene family and stress responses. For instance, in sweet potato, a CAD gene transcript has been found to be highly induced by cold (Kim et al., 2010b). Our observation of the up-regulated CAD protein in petunia seedlings at 2°C offers another line of evidence for this relationship, implicating positive translational regulation of lignin biosynthesis as part of the petunia response under cold stress conditions. The resultant change in lignin content may alter water permeability and/or CW rigidity in cold-exposed petunia plantlets and, thereby, influence their ability to cope with the adverse cold conditions. Expansins are plant CW-remodeling proteins. The published data demonstrate that they are mainly involved in the pH-dependent extension of plant CWs and expansins are also involved in CW modifications and cell enlargement induced by plant hormones. Interestingly, the expansin-like gene *EXLA2* has been reported to be induced by cold and salinity, as well as by abscisic acid (ABA) treatment. Furthermore, the *exla2* mutant exhibited a hypersensitive response to increased cold and salt which was mediated by ABA (Abuqamar et al., 2013). Along with other reports (Abuqamar, 2014), the data indicate that it is not unreasonable to consider the possibility that certain expansins contribute significantly to abiotic stress responsiveness and impact signaling pathways that regulate gene expression. Even though the precise role of these two CW related proteins under low temperature stress is still obscure, future detailed analysis of them at the molecular, biochemical and physiological levels may provide an insight into the signaling pathway involved in the regulation of cold stress adaption.

In addition, within the list of cold-responsive proteins, a dehydrin (E5F371) and a methionine aminopeptidase (K4ASP6) were identified. Dehydrins, also known as the D-11 family of late embryogenesis abundant proteins, are formed during stresses which cause dehydration of the cells such as drought, salinity, heat and cold. The expression of dehydrins is closely related with cold resistance in numerous plant species such as *Arabidopsis* (Kawamura and Uemura, 2003), *Citrus* (Hara et al., 2003), strawberry (Houde et al., 2004), rice (Lee et al., 2005b), and *Rhododendron* (Peng et al., 2008), and such accumulated dehydrins are believed to be key components of the cold acclimation process. Consistent with these findings, we identified a dehydrin candidate which was up-regulated almost 2.3-fold in cold-treated lines when compared with controls. In *Arabidopsis*, the levels of some dehydrins have been reported to be regulated by CBF transcription factors of the cold-response pathway (Lee et al., 2005a). However, it is noteworthy that the expression of individual dehydrin proteins may exhibit particular patterns of tissue specificity which vary according to the different plant species (Koehler et al., 2012) and, thus, it may be useful to conduct further studies of this dehydrin in Petunia. Methionine aminopeptidases (MAPs) are well-known to be required for correct plant development, as demonstrated in *Arabidopsis* (Ross et al., 2005). It seems to have no relevance to cold stress resistance in plants. However, a recent study of the barley DNA-binding MAP, whose localization changes from the nucleus to the cytoplasm under low temperature treatments, suggested that this novel MAP could also function in conferring freezing tolerance by facilitating protein maturation (Jeong et al., 2011). This result leads us to tentatively suggest that the responsiveness of the petunia MAP to cold stress, observed in our study here, might not be an incidental response, but may deserve further investigation.

In summary, this work offers a global perspective of the petunia proteomic profile under cold stress, achieved through the use of the iTRAQ technique. A total of 117 cold-responsive proteins were revealed which are therefore potential candidates to be involved in the overall plant response aimed at achieving the beneficial equilibrium of physiological homeostasis. Our study provides not only novel insights into the plant response to cold stress, but also a promising starting point for further investigations into the functions of candidate proteins as part of the petunia cold response. Nevertheless, it should be noted that a significant proportion of the revealed DEPs remained unidentified with respect to probable function, in large part due to the lack of high quality functional annotations for many plant genomes. The experimental validation of these un-annotated proteins will make an important contribution to bridge the gap between proteomic discoveries of stress-responsive proteins and the selection of target proteins with strong potentiality for the improvement of cold tolerance by genetic engineering in plants (Gong et al., 2015).

## Author contributions

WZ and MB designed the experiments. WZ, HZ, LN, and BL performed the experiments. WZ analyzed the data and drafted the manuscript. MB thoroughly revised the manuscript and finalized the manuscript. All the authors read and approved the manuscript.

### Conflict of interest statement

The authors declare that the research was conducted in the absence of any commercial or financial relationships that could be construed as a potential conflict of interest.

## Supplementary material

Figure S1 **Evaluation of samples by SDS-PAGE analysis**.

Figure S2 **Fractionation of combined iTRAQ labeled peptides**.

Figure S3 **High variance of protein quantification**.

Table S1 **Primers used for real-time RT-PCR analysis**.

Table S2 **Protein concentration of samples determined by BCA**.

Table S3 **Quantification of digested peptides abundance**.

Table S4 **Quantification of total unique peptides**.

Table S5 **Identification of all detected proteins**.

Table S6 **Relative quantification and significance analysis of proteins detected in each replicate of all samples**.

Table S7 **GO enrichment analysis**.

Table S8 **Prediction of subcellular localization of DEPs**.
