# Supplementary figures and images for: Quantitative Proteomic Analysis Provides Novel Insights into Cold Stress Responses in Petunia Seedlings
Source: Front Plant Sci. 2016 Feb 25;7:136. doi: 10.3389/fpls.2016.00136 (PMC4766708; doi:10.3389/fpls.2016.00136)

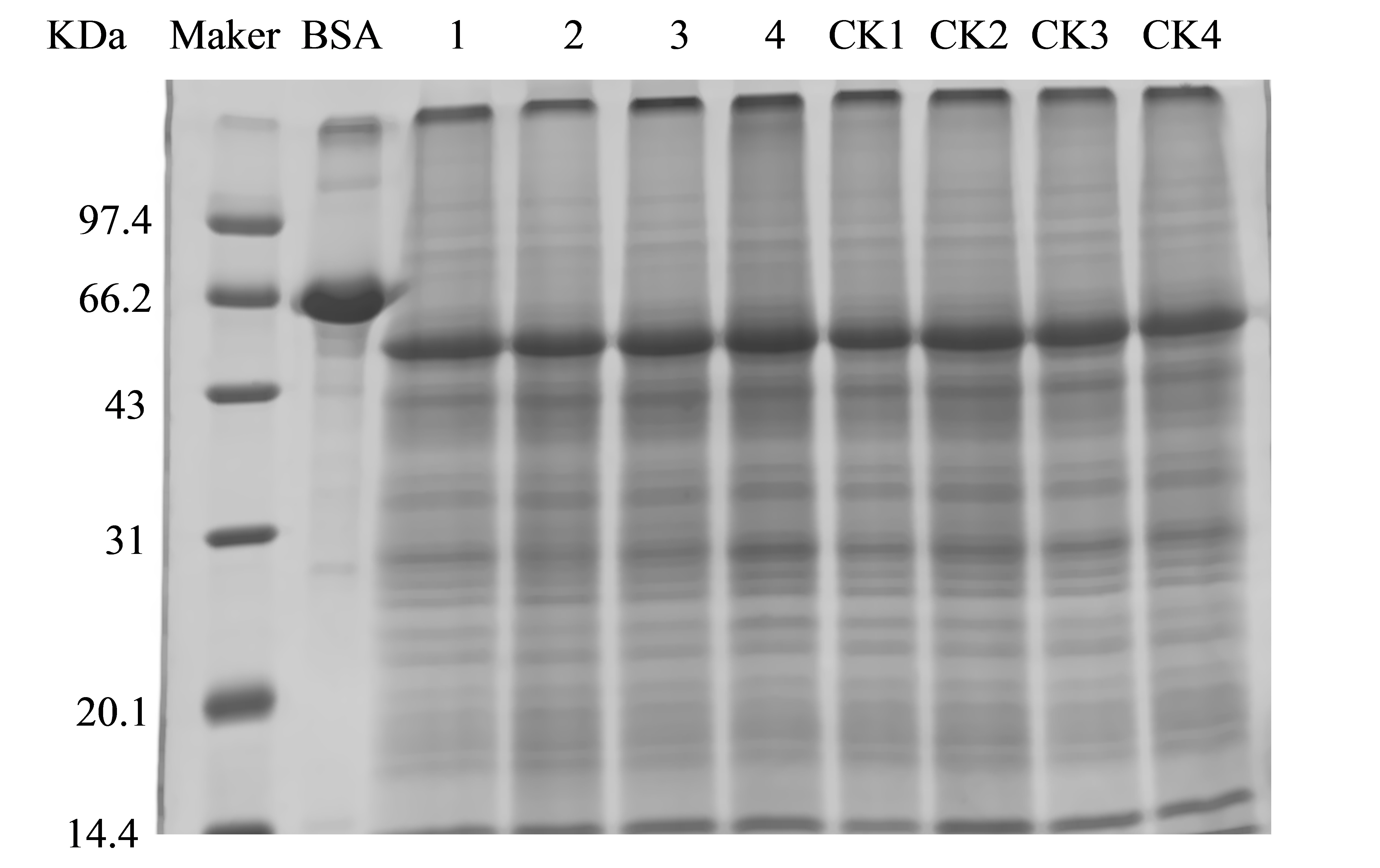

Supplement: Figure S1 — Evaluation of samples by SDS-PAGE analysis. [file Image1.JPEG]

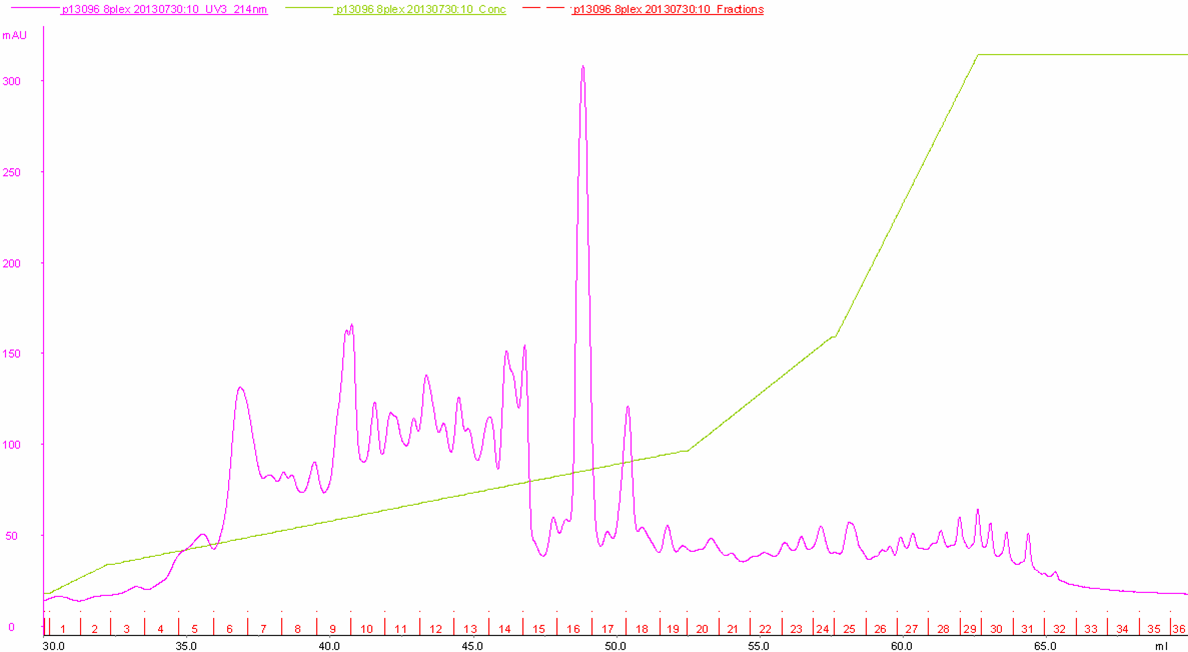

Supplement: Figure S2 — Fractionation of combined iTRAQ labeled peptides. [file Image2.PNG]

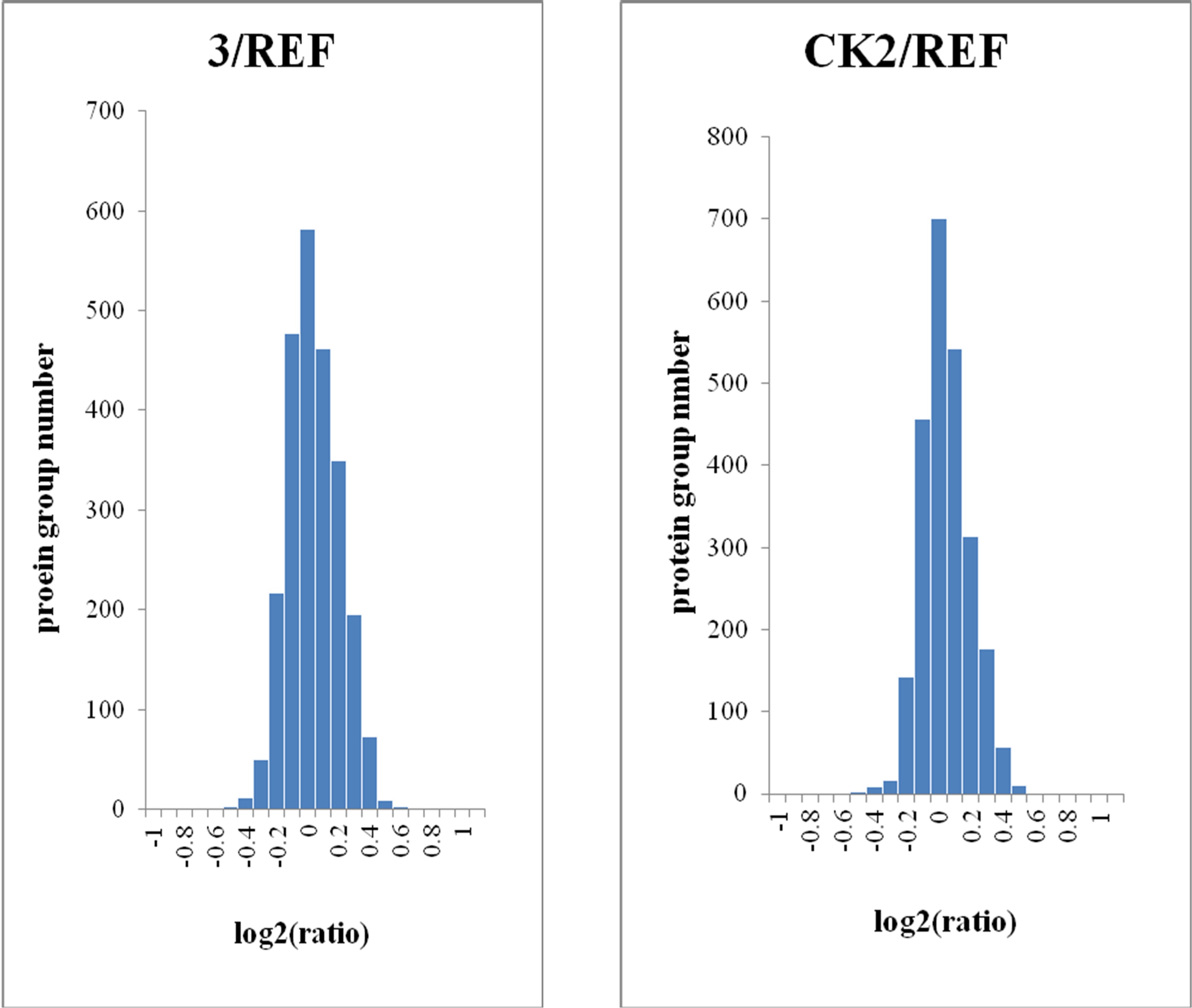

Supplement: Figure S3 — High variance of protein quantification. [file Image3.JPEG]
