# Supplementary material for: Quantitative Proteomic Analysis Provides Novel Insights into Cold Stress Responses in Petunia Seedlings
Source: Front Plant Sci. 2016 Feb 25;7:136. doi: 10.3389/fpls.2016.00136 (PMC4766708; doi:10.3389/fpls.2016.00136)
Supplement: Table S2 — Protein concentration of samples determined by BCA. [file Table2.DOC]

**Table S2. Protein concentration of samples determined by BCA**

| Sample | 1 | 2 | 3 | 4 | CK1 | CK2 | CK3 | CK4 |
| --- | --- | --- | --- | --- | --- | --- | --- | --- |
| Concentration (μg/μL) | 8.2 | 8.8 | 11.8 | 6.2 | 10.4 | 9.7 | 6.8 | 4.9 |
