# Supplementary material for: Quantitative Proteomic Analysis Provides Novel Insights into Cold Stress Responses in Petunia Seedlings
Source: Front Plant Sci. 2016 Feb 25;7:136. doi: 10.3389/fpls.2016.00136 (PMC4766708; doi:10.3389/fpls.2016.00136)
Supplement: Table S3 — Quantification of digested peptides abundance. [file Table3.DOC]

**Table S3 Quantification of digested peptides abundance**

| Sample | 1 | | 2 | | 3 | | 4 | | CK1 | | CK2 | | CK3 | | CK4 | |
| --- | --- | --- | --- | --- | --- | --- | --- | --- | --- | --- | --- | --- | --- | --- | --- | --- |
| Concentration (μg/μL) | | 1.4 | | 1.4 | | 1.4 | | 1.6 | | 2.3 | | 1.3 | | 1.3 | | 1.4 |
